# Understanding the role of soluble proteins and exosomes in non-invasive urine-based diagnosis of preeclampsia

**DOI:** 10.1038/s41598-024-75080-2

**Published:** 2024-10-15

**Authors:** Taewoon Kim, Harshitha Kallubhavi Choodinatha, Kwang Sik Kim, Kyusoon Shin, Hyeon Ji Kim, Jee Yoon Park, Jong Wook Hong, Luke P. Lee

**Affiliations:** 1 Department of Bionanotechnology, Graduate School, Hanyang University, Seoul, 04763 Korea; 2 Department of Obstetrics and Gynecology, Seoul National University College of Medicine, Seoul, Korea; 3 Seoul National University, Seoul, Korea; 4 Department of Obstetrics and Gynecology, Seoul National University College of Medicine, Seoul National University Bundang Hospital, Seongnam, Korea; 5 Department of Medical and Digital Engineering, Graduate School, Hanyang University, Seoul, 04763 Korea; 6 Department of Bionanoengineering, Hanyang University, 15588 Ansan, Gyeonggi-do Korea; 7 Harvard Medical School, Department of Medicine, Harvard University, Brigham and Women’s Hospital, Boston, MA USA; 8 Department of Bioengineering, University of California at Berkeley, Berkeley, CA USA; 9 Department of Electrical Engineering and Computer Science, University of California at Berkeley, Berkeley, CA USA; 10 Department of Biophysics, Institute of Quantum Biophysics, Sungkyunkwan University, Suwon, Korea; 11 Department of Chemistry & Nanoscience, Ewha Womans University, Seoul, Korea

**Keywords:** Preeclampsia, Pregnancy complications, Non-invasive diagnosis, SFlt-1/PlGF ratio, Urine, Urinary exosome, ELISA, Diagnostic markers

## Abstract

Preeclampsia is a hypertensive disorder of pregnancy that can lead to stillbirth and preterm birth if not treated promptly. Currently, the diagnosis of preeclampsia relies on clinical symptoms such as hypertension and proteinuria, along with invasive blood tests. Here, we investigate the role of soluble proteins and exosomes in noninvasive diagnosing preeclampsia non-invasively using maternal urine and urine-derived exosomes. We quantified the levels of particles and the presence of TSG101 and CD63 in urine and urinary exosomes via the biologically intact exosome separation technology (BEST) platform. Then, we obtained higher levels of soluble proteins such as fms-like tyrosine kinase-1 (sFlt-1) and placental growth factor (PlGF) from urine as it was than urinary exosomes. Compared to commercial blood tests, the sensitivity of the sFlt-1/PlGF ratio was found to be 4.0 times higher in urine tests and 1.5 times higher in tests utilizing urine-derived exosomes. Our findings offer promising possibilities for the early and non-invasive identification of high-risk individuals at risk of preeclampsia, allowing for comprehensive preventive management.

**Supplementary Information:**

The online version contains supplementary material available at 10.1038/s41598-024-75080-2.

## Introduction

Preeclampsia is a severe complication of pregnancy characterized by new onset high blood pressure, often associated with proteinuria and damage to multi-organs, predominantly the liver, kidney, lungs, and brain^1–3^. Symptoms of preeclampsia include hypertension, headaches, visual disturbances, and edema involving feet, hands, and face^4^. Preeclampsia is diagnosed after 20 weeks of pregnancy. If untreated, preeclampsia can lead to severe complications, including preterm birth, fetal growth restriction, placental abruption, disseminated intravascular coagulation, pulmonary edema, long-term consequences like cardiovascular diseases, chronic kidney disease, and even mortality for both mother and fetus^5–8^.

The classical criteria to diagnose preeclampsia is based on clinical symptoms such as hypertension and proteinuria. However, these manifestations are not exclusive to preeclampsia, and some patients with preeclampsia do not exhibit noticeable symptoms until the very late period of gestation^9^. Since a few years ago, a test measuring soluble fms-like tyrosine kinase-1 (sFlt-1) and placental growth factor (PlGF) from maternal blood has been introduced and is now widely used in clinical practice to predict the development of preeclampsia^10–12^. The two markers originate from the placenta and vascular structure and affect placental vascular growth during pregnancy. sFlt-1 inhibits angiogenesis, while PlGF promotes it. It is known that sFlt-1 binds to PlGF and vascular endothelial growth factor (VEGF), thereby preventing their interaction with endothelial cell surface receptors^13^. This binding reduces the availability of free PlGF, consequently inhibiting angiogenesis within the placenta and leading to the development of preeclampsia^14,15^. Both the concentrations of sFlt-1 and PlGF are related to the onset and severity of preeclampsia. Among women with suspected preeclampsia, the sFlt-1/PlGF ratio has a very high negative predictive value of ruling out the development of preeclampsia within 7 days, adverse maternal outcomes within 14 days, or delivery with preeclampsia within 14 days^16^. In May 2023, the U.S Food and Drug Administration approved the use of The B·R·A·H·M·S sFlt-1/ PlGF KRYPTOR Test System to be used alongside other laboratory tests and clinical assessments to aid in the risk assessment of pregnant women for progression to preeclampsia with severe features within 2 weeks of presentation (https://www.fda.gov/news-events/press-announcements/fda-roundup-may-19-2023). Still, the commercially available test has certain limitations, such as invasiveness since maternal blood should be obtained and low accessibility that the patients have to visit the institution with the appropriate equipment for the test.

Recently, as a non-invasive approach to diagnosing preeclampsia, ongoing attempts have been made to measure various biomarkers such as sFlt-1, PlGF, and soluble endoglin (sEng) with maternal urine samples^17–19^. Additionally, research projects aiming to diagnose preeclampsia through exosomes isolated from blood and urine have been reported to enhance accuracy and sensitivity^20–23^. These studies utilized exosome-derived biomolecules, such as proteins, lipids, and nucleic acids, taking advantage of exosomes containing genetic information from their parent cells^24,25^. Furthermore, since exosomes are found in various body fluids, including tears, urine, saliva, as well as blood, they hold promising potential as a non-invasive diagnostic tool and for the advancement of treatments^26–29^. However, exosomes isolated by ultracentrifugation can undergo damage during the separation process, potentially leading to decreased diagnostic accuracy^30,31^. Therefore, it is crucial to develop an efficient exosome isolation method that minimizes damage, ensuring reliable diagnostics for preeclampsia.

In this study, we aim to compare the use of urine and intact urine-derived exosomes from patients with preeclampsia and healthy pregnant women for diagnosing preeclampsia. We analyzed the characteristics of these exosomes and measured the concentrations of sFlt-1 and PlGF within them. Furthermore, we compared the sFlt-1/PlGF ratio in blood, urine, and urinary exosomes to investigate their relationship.

## Results

### Pathogenesis of preeclampsia and diagnostic approach using biomarkers

**Figure 1:**
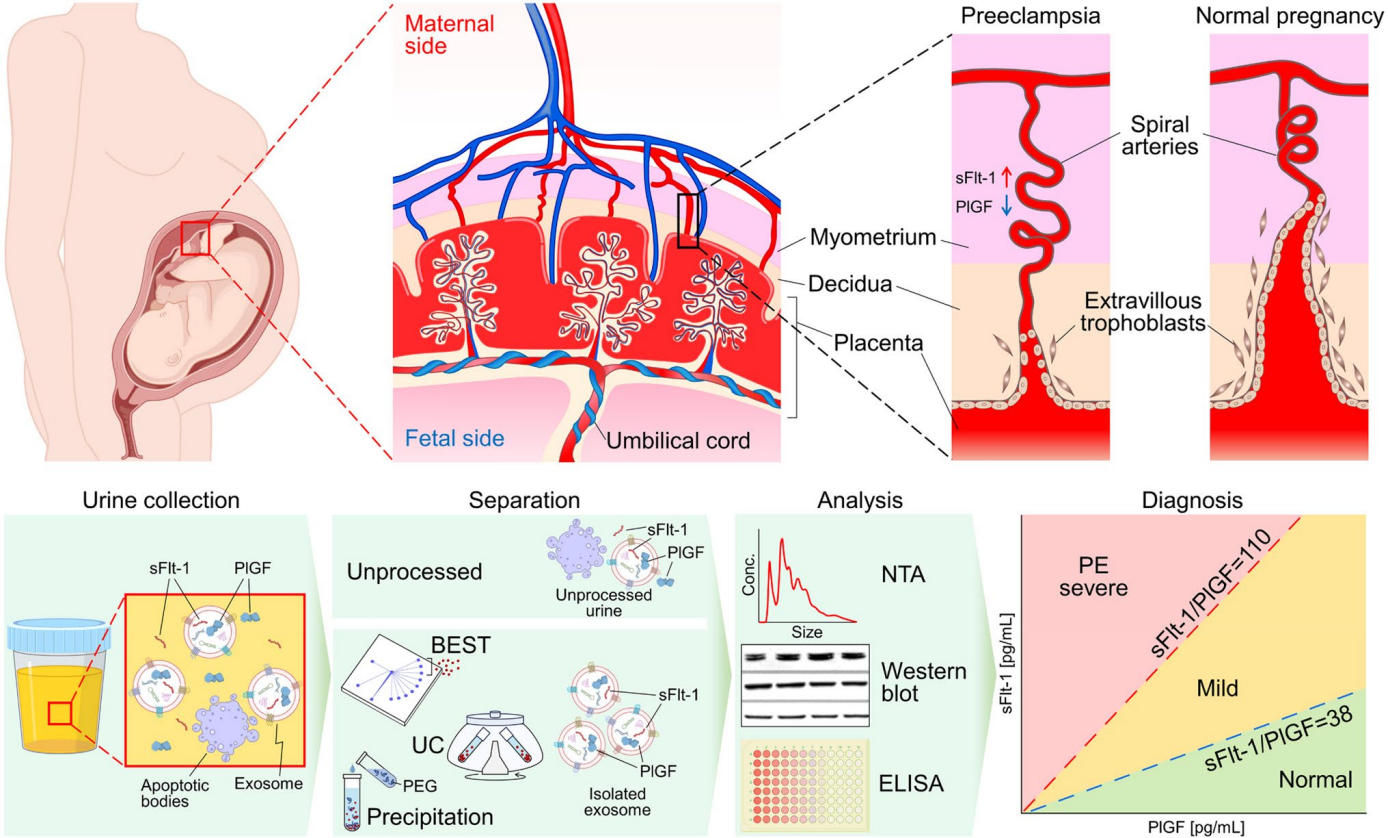
Pathogenesis of preeclampsia and diagnostic process. Failure of trophoblast interaction in early pregnancy results in a stress response in the placenta that affects villous growth and development. It negatively affects the delivery of sufficient oxygen and nutrients to the fetus and releases anti-angiogenic factors, leading to multi-organ dysfunction. We utilized Biologically intact Exosome Separation Technology (BEST) to isolate exosomes from urine samples of patients with preeclampsia and healthy controls. We further characterize the isolated urinary exosomes and urine to facilitate the diagnosis of preeclampsia. sFlt-1, soluble fms-like tyrosine kinase 1; PlGF, placental growth factor; UC, ultracentrifugation; PEG, polyethylene glycol.

Preeclampsia has a complex pathophysiology, and the precise etiology remains unclear. However, placental dysfunction and oxidative stress are recognized factors in its development^32,33^. As shown in Fig. 1, during normal pregnancy, the trophoblasts that comprise the placenta invade the inner third of the myometrium to form villi, a network of fine projections^34^. Villi are twig-like structures that absorb oxygen and nutrients from the maternal blood vessels and deliver them to the developing fetus. However, in the case of pregnant women with preeclampsia, invasion of the extravillous trophoblast into the uterine decidua is inefficient and superficial, resulting in incomplete spiral artery remodeling. This causes chronic placental ischemia and reduced blood flow to the developing fetus^35^. Several epidemiologic studies have demonstrated that placental oxidative stress causes decreased levels of PlGF, elevated levels of sFlt-1, or increased sFlt-1/PlGF ratio in serum^36,37^. This suggests that changes in specific markers are linked with the development of the disease in patients with preeclampsia^38–40^. Accordingly, in this study, we evaluated the possibility of diagnosing preeclampsia using sFlt-1 and PlGF obtained from urine and urine-derived exosomes.

### Characterization of urine and urine-derived exosomes in patients with preeclampsia and healthy controls

**Figure 2:**
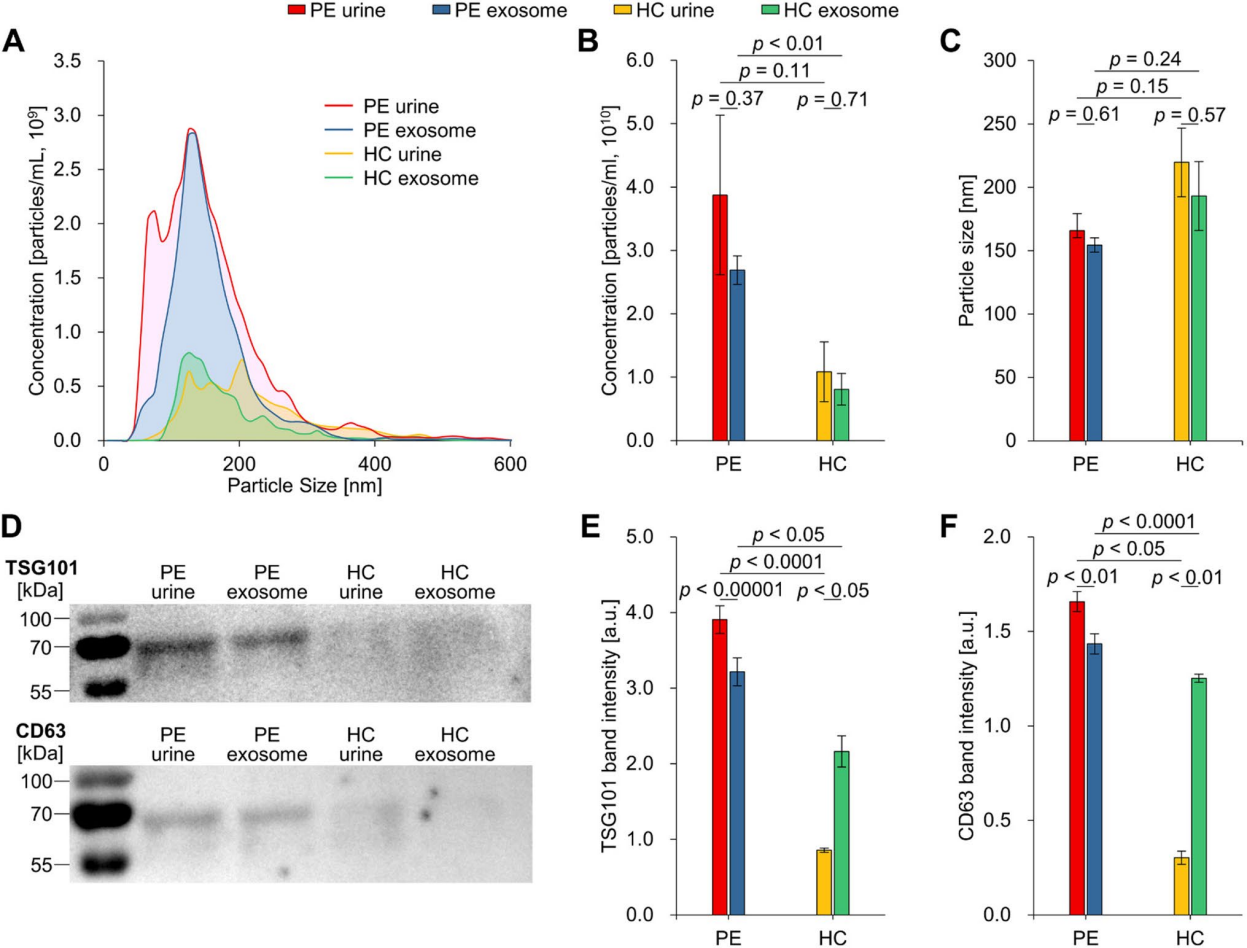
Characterization of urine and urinary exosomes isolated by BEST of PE and HC. (**A**) Representative nanoparticle tracking analysis for untreated urine and isolated urine from PE and HC, depicting particle diameter and the number of particles. (**B**) and (**C**) Comparisons of particle concentration and particle size between PE and HC. (**D**) Western blots of TSG101 and CD63 protein extracted from isolated exosomes. (**E**) and (**F**) Quantitative analysis of exosome marker TSG101 and CD63 protein. All data are presented as mean $\:\pm\:$ standard error and analyzed by paired t-test and independent two-tailed t-test. BEST, biologically intact exosome separation technology; PE, patients with preeclampsia; HC, healthy controls.

In our study, we recruited a total of 6 pregnant women, including three healthy mothers as the control group (HC) and three mothers diagnosed with preeclampsia (PE). As depicted in Fig. 1, each participant provided a urine sample, and exosomes were isolated using biologically intact exosome separation technology (BEST). The particle size distribution and particle concentration of urine and urine-derived exosomes from PE and HC are shown in Fig. 2A–C. The PE and HC particle concentrations were 2.7 × 10^9^ and 8.1 × 10^8^ particles/mL, respectively. Furthermore, as demonstrated in Fig. 2D–F, we confirmed the identity of the isolated particles as exosomes by utilizing the exosome markers TSG101 and CD63 (Supplementary Fig. S2). The expression levels of these markers were higher in PE compared to HC. The elevated protein expression levels in PE suggest the presence of altered exosome composition associated with the disease state, indicating a potential role for exosomes in this pathological condition.

### Efficiency of exosome isolation techniques for preeclampsia diagnosis

**Figure 3:**
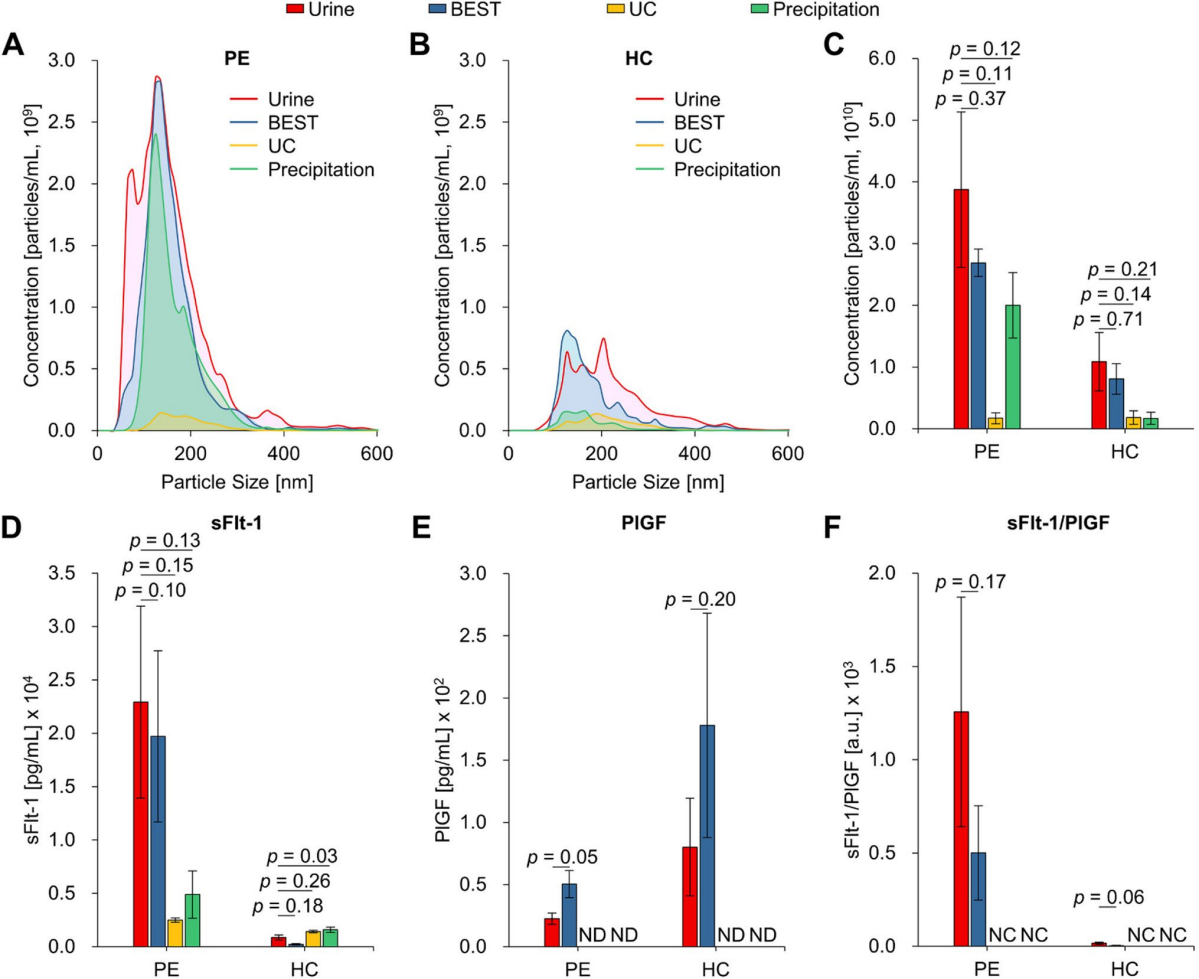
Comparison of exosome-derived marker signals according to separation methods. (**A**) and (**B**) Nanoparticle tracking analysis for untreated urine and urinary exosomes isolated from BEST, UC, and precipitation of PE and HC, depicting particle diameter and the number of particles. (**C**) Comparisons of particle concentration between PE and HC. (**D**)-(**F**) Comparison of sFlt-1, PlGF, and sFlt-1/PlGF ratio obtained from untreated urine and urinary exosomes isolated from BEST, UC, and precipitation. All data are presented as mean $\:\pm\:$ standard error and analyzed by paired t-test. BEST, biologically intact exosome separation technology; UC, ultracentrifugation; PE, patients with preeclampsia; HC, healthy controls; sFlt-1, soluble fms-like tyrosine kinase 1; PlGF, placental growth factor; ND, not detected; NC, not calculated.

To assess the separation efficiency of different exosome isolation techniques, we incorporated ultracentrifugation (UC) and precipitation methodologies as part of our comparative analysis. As shown in Fig. 3A–C, an evaluation of the size distribution and particle concentration in exosomes isolated through these various methods was conducted. Notably, there were no statistically significant differences in particle concentration observed among the samples derived from urine, BEST, UC, and precipitation. Nevertheless, noteworthy findings emerged when examining samples from PE and HC. Within these subsets, our BEST methodology outperformed the others, delivering the highest particle concentration of 2.7 × 10^10^ particles/mL. Precipitation came in second with 2.0 × 10^10^ particles/mL, while UC exhibited the lowest concentration, measuring at 1.7 × 10^9^ particles/mL. In addition to particle concentration assessments, we also analyzed the sFlt-1 and PlGF levels for each method, as shown in Fig. 3D–F. In PE, we observed that sFlt-1 concentrations exhibited a trend similar to particle concentration. However, it’s crucial to note that the PlGF concentration was insufficient for accurate measurement with UC and precipitation methods, rendering them inadequate for calculating the sFlt-1/PlGF ratio. Consequently, our ability to determine the sFlt-1/PlGF ratio was restricted to urine samples and exosomes isolated exclusively through the BEST method.

### Quantification and comparative analysis of sFlt-1 and PlGF concentrations in urine and urine-derived exosomes

**Figure 4:**
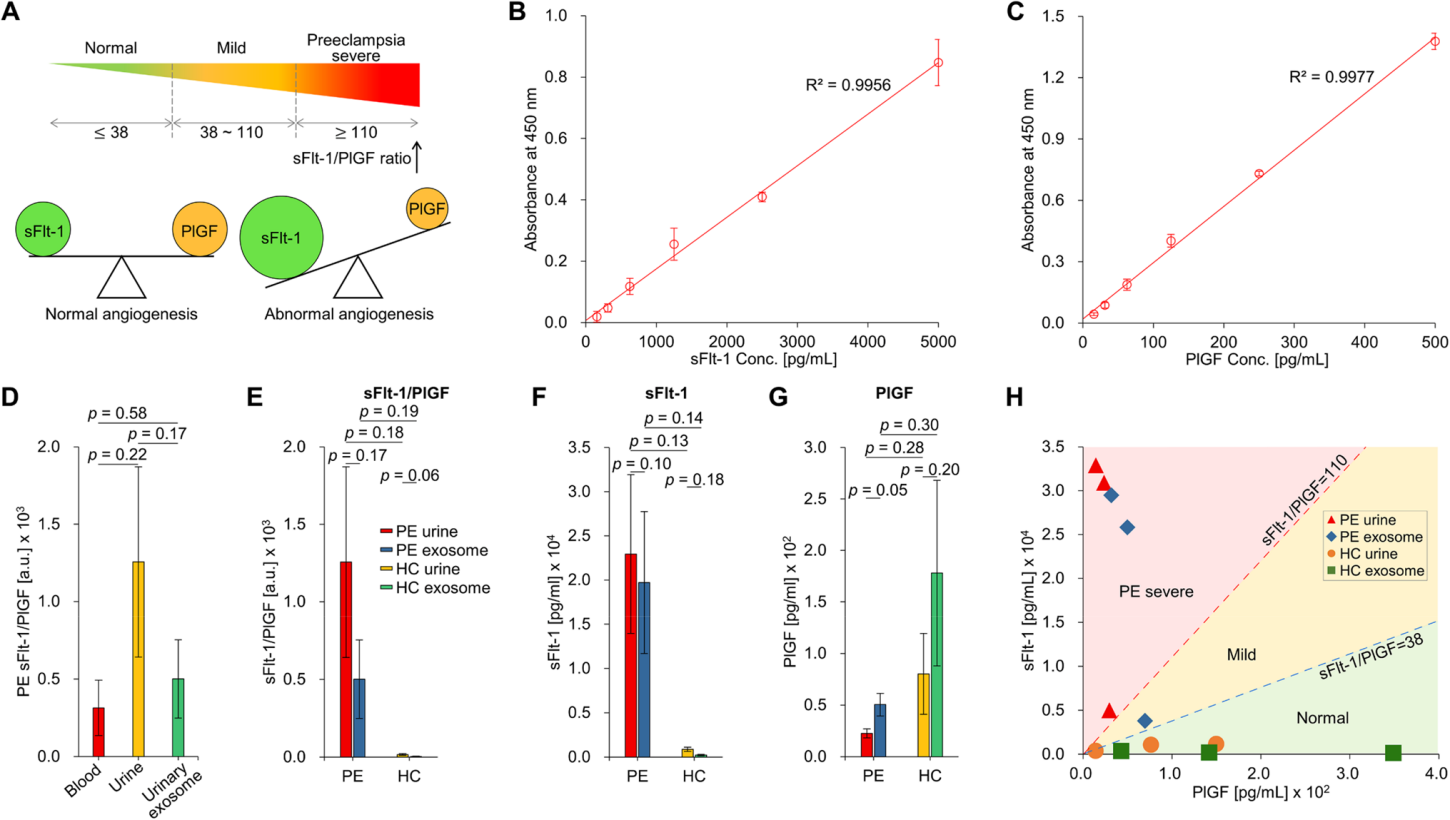
Quantification of sFlt-1 and PlGF concentrations in urine samples. (**A**) Clinical characteristics of preeclampsia according to sFlt-1 and PlGF concentrations and sFlt-1/PlGF ratio. (**B**)-(**C**) Standard curves for sFlt-1 and PlGF ELISA. Target protein concentration in urine samples was determined using the standard curve. (**D**) Comparison of sFlt-1/PlGF ratio in blood, urine, and urine-derived exosomes of PE. The sFlt-1/PlGF ratio was higher in urine and urine-derived exosomes than in blood. (**E**)-(**G**) Comparison of sFlt-1, PlGF, and sFlt-1/PlGF ratio obtained from urine and urinary exosomes of PE and HC. (**H**) Assessment of predictive performance of the sFlt-1/PlGF ratio for diagnosing preeclampsia using urinary exosomes. The blue and red dotted line represents the sFlt-1/PlGF ratio cutoff of 38 and 110, respectively. All data are presented as mean $\:\pm\:$ standard error and analyzed by paired t-test and independent two-tailed t-test. PE, patients with preeclampsia; HC, healthy controls; sFlt-1, soluble fms-like tyrosine kinase 1; PlGF, placental growth factor.

As shown in Fig. 4A, preeclampsia can be diagnosed by evaluating the ratio of sFlt-1 to PlGF, which is determined through a specific blood test^41^. An increased ratio (sFlt-1/PlGF ratio ≥ 38) typically indicates an increased risk of preeclampsia^16,42^. Therefore, we measured and compared the sFlt-1/PlGF ratio between PE and HC. The results in Fig. 4D demonstrated that the average sFlt-1/PlGF ratio in PE was 314 in blood, 1256 in urine, and 501 in urine-derived exosomes, which were 4.0 times and 1.6 times higher, respectively. These findings imply that utilizing urine and urine-derived exosomes as diagnostic markers may significantly improve sensitivity in detecting preeclampsia, owing to the notably elevated sFlt-1/PlGF ratio observed in these samples.

In addition, we assessed the diagnostic significance of sFlt-1 and PlGF by comparing their concentrations in urine and urine-derived exosomes and analyzing the sFlt-1/PlGF ratio. The sFlt-1/PlGF ratio is presented in Fig. 4E, showing an average value of 16.4 in HC and 1256.2 in PE for urine, indicating a higher value than that of HC. Similarly, the average sFlt-1/PlGF ratio in urine-derived exosomes was 3.4 in HC and 500.6 in PE, representing lower values compared to urine. Figure 4F and G demonstrated higher levels of sFlt-1 in PE than in HC, while the concentration of PlGF was higher in HC than in PE. Moreover, Fig. 4H showed that each mother with preeclampsia had sFlt-1/PlGF values of 38 or higher, whereas all values in the control group were below 38. This indicates that not only blood but also urine and urine-derived exosomes can be used for the diagnosis of preeclampsia.

## Discussion

In recent years, biomarkers such as PlGF, sFlt-1, and sEng have been extensively studied and validated for predicting preeclampsia, demonstrating strong predictive performance in early-onset cases^11,43^. Based on this foundation, our study investigated the potential of using urine and urine-derived exosomes as non-invasive diagnostic tools. We focused on analyzing the sFlt-1/PlGF ratio in these samples to assess its effectiveness in diagnosing preeclampsia. Our findings suggest that these biomarkers measured in urine and exosomes can provide valuable diagnostic insights, potentially offering an accessible alternative to invasive methods.

Our study initially hypothesized that utilizing exosomes isolated from urine could provide a higher sensitivity in diagnosing preeclampsia than urine^44,45^. However, contrary to our expectations, our results demonstrated that urine-soluble proteins exhibited a higher sensitivity than urine-derived exosomes for diagnosing preeclampsia. Several factors might contribute to this unexpected outcome. First, it’s essential to consider the complexity of the pathophysiology of preeclampsia^34,46^. Preeclampsia is a multifaceted disorder with intricate mechanisms involving various biological processes and markers. While sFlt-1 and PlGF are critical markers for diagnosing preeclampsia^47,48^, the biomolecule composition of exosomes may be different from that of urine or serum, resulting in low sensitivity. Urine itself with soluble proteins, on the other hand, is a direct and comprehensive source of a wide array of biomarkers, making it a more sensitive specimen for detecting physiological deviations associated with preeclampsia^49^.

Additionally, isolating exosomes might introduce variations or alterations in their composition. Notably, sFlt-1 and PlGF are soluble proteins and not membrane proteins found within exosomes^50^. Due to their soluble nature, a substantial amount of sFlt-1 and PlGF may be inadvertently removed during the isolation process, potentially leading to a weakened ELISA signal. As shown in Fig. 3, techniques utilized for exosome isolation could impact the integrity of exosomes and the biomolecules they carry^51^. These alterations affect the accuracy and sensitivity of the diagnostic readouts. Furthermore, more than the abundance and purity of the exosomes obtained from urine might be required to provide a higher sensitivity than the raw urine sample. The isolation process may inadvertently lead to loss or dilution of critical exosome-associated biomarkers, thereby compromising the sensitivity of the diagnostic test based on exosomal content. Moving forward, it is imperative to refine the methods of exosome isolation and characterization, exploring alternative isolation techniques that could preserve the integrity and composition of exosomes more effectively. Moreover, a more in-depth investigation into the specific biomolecular content of both urine and urine-derived exosomes is warranted to identify potential markers that might contribute to the higher sensitivity observed in the raw urine and urine-derived exosome.

Despite the potential benefits demonstrated in the study, it is still considered preliminary due to the small sample size, which included a limited number of patients with preeclampsia and controls. This limited small sample size is a primary factor contributing to the high standard errors observed in our data, as it may need to capture the variability present in a larger population fully. Furthermore, noticeable variability, as indicated by high standard errors, was observed in the sFlt-1 and PlGF values obtained from urine and urine-derived exosomes. This variability can be attributed to inherent biological differences among individuals, especially given the complexity of preeclampsia^52^. The natural variation in biomarker concentrations, such as sFlt-1 and PlGF, among individuals can further increase the standard error in the measured values^53^. To address this, we emphasize that increasing the sample size is crucial. Incorporating a larger number of samples provides a more comprehensive representation of exosome compositions, reducing the impact of outliers and extreme variations often present in smaller sample sets. A diverse sample set better mirrors population heterogeneity, enhancing the generalizability of our findings.

Moreover, it’s crucial to acknowledge the inherent differences between urine and serum as biological fluids^54^. These differences can impact the performance and reliability of the ELISA kit when applied to urine and urine-derived exosomes. To validate the applicability of the serum-based diagnostic criteria for preeclampsia in urine and urine-derived exosomes, a comprehensive cohort study with a larger sample size is imperative. This expanded cohort should specifically focus on pregnant individuals, both with and without preeclampsia, and involve rigorous testing utilizing the ELISA kit optimized for urine samples. Comparing the results obtained from serum and urine analyses will provide critical insights into the potential variations in biomarker levels and ratios between these two biological fluids. It will also shed light on whether the established diagnostic thresholds for preeclampsia in serum can be appropriately extrapolated to urine and urine-derived exosomes.

Furthermore, exploring alternative biomarkers that are more specific to urine and exosomal compositions could enhance the diagnostic accuracy for preeclampsia. By doing so, it may be possible to augment the sensitivity of preeclampsia diagnosis using exosomes, enabling early detection before 20 weeks of gestation. Consequently, further research efforts should prioritize discovering and validating novel biomarkers to develop more accurate and reliable diagnostic methods for preeclampsia.

In conclusion, our study has shed light on a potential breakthrough in preeclampsia diagnosis. By evaluating the concentrations of sFlt-1 and PlGF in urine and urine-derived exosomes, we have demonstrated the feasibility of utilizing the sFlt-1/PlGF ratio in urine and urine-derived exosomes as a diagnostic tool for preeclampsia. These findings suggest that urine samples hold promise as a non-invasive and easily accessible source of crucial diagnostic information, enhancing the accuracy of preeclampsia diagnosis. If further validated and refined, this approach could revolutionize how we detect and manage this serious pregnancy complication, potentially leading to improved maternal and fetal outcomes.

## Methods

### Ethics statement

This study was approved by the Seoul National University Bundang Hospital Institutional Review Board (B-1904-537-304) and written informed consent was obtained from all participants. We confirmed that all experiments were performed in accordance with relevant guidelines and regulations.

### Urine sample preparation

We obtained about 10 cc of urine samples from pregnant women with more than 24 weeks of gestational age who had been admitted to Seoul National University Bundang Hospital. Three samples were from pregnant women diagnosed as preeclampsia and for control, three healthy cases were selected. Preeclampsia was diagnosed when the pregnant woman presented with hypertension and more than one of the following features of preeclampsia: proteinuria (≥ 300 mg per 24-hour urine collection [or this amount extrapolated from a timed collection], or protein: creatinine ratio ≥ 0.3, or urine dipstick reading ≥ 2+ [if other quantitative methods are not available]), thrombocytopenia (platelet count < 100,000/microL), renal insufficiency (serum creatinine of > 1.1 mg/dL or a doubling of the serum creatinine concentration in the absence of other renal disease), impaired liver function as indicated by liver transaminase levels at least twice the normal concentration, pulmonary edema, persistent cerebral or visual symptoms^55^. During pregnancy, hypertension is defined as systolic blood pressure ≥ 140 mmHg and/or diastolic blood pressure ≥ 90 mmHg. After collection, the urine samples were stored at -80 °C until exosome separation. The separation of exosomes was performed using biologically intact exosome separation technology (BEST), which has been previously reported^56^. For exosome isolation, the urine samples were immediately injected into the sample channel after being melted at 37 °C for 3 min. Dulbecco’s phosphate-buffered saline (DPBS, SolBio, Republic of Korea) was injected into the buffer channel. The flow rate of sample: buffer: suction was set to 5:95:75, as shown in Supplementary Fig. 1A.

### Exosome isolation using ultracentrifugation (UC)

Ultracentrifugation was performed using a Beckman Coulter Optima XE-100 Ultracentrifuge. A 5 mL of urine was centrifuged at 2,000 g for 10 min to remove cell debris and large vesicles. The supernatant was then centrifuged at 100,000 x g for 90 min twice to pellet exosomes using Beckman Coulter SW32Ti rotor. The supernatant was removed and the exosome pellet was resuspended in 500 µL of DPBS^44,57^, as shown in Supplementary Fig. 1B.

### Exosome isolation using polyethylene glycol (PEG)

A 5 mL of urine sample was centrifuged at 3,000 g for 15 min to remove cells and cell debris. The supernatant was transfer to a sterile vessel, and then mixed with a ExoQuick solution (System Biosciences, Inc., USA). After that, the mixture was incubated at 4℃ for at least 12 h, and then the incubated solution was centrifuged at 1,500 g for 30 min. The supernatant was aspirated, and the exosome pellet was resuspended in 500 µL of DPBS^58,59^, as shown in Supplementary Fig. 1C.

### Nanoparticle tracking analysis (NTA)

Nanoparticle tracking analysis was employed to assess the size distribution and particle concentration of the isolated exosomes. NTA measurements were conducted using the NanoSight LM10 system (Malvern Instruments Ltd., UK). Prior to analysis, the sample chamber was cleaned with ethanol to ensure accurate measurements. The exosomes were then appropriately diluted in DPBS to achieve a concentration that allowed for the examination of 20 particles per frame. Subsequently, 500 µL of the diluted sample was injected into the sample chamber of the NanoSight LM10 instrument, which was equipped with a 642 nm laser. The Brownian motion of the particles was recorded using a camera, and the NanoSight NTA software (Ver. 3.1 build 3.1.46, https://www.malvernpanalytical.com/en) was utilized to analyze the particle movement, determine the size distribution, and calculate the particle concentration. Each sample underwent three separate measurements, with each analysis lasting 30 s. To ensure accurate and reliable results, the sample chamber was cleaned with clean deionized water between each measurement.

### Western blot (WB)

Western blot analysis was conducted to detect specific exosomal markers, including TSG101 and CD63^60,61^. For this purpose, exosomes were lysed using radioimmunoprecipitation assay (RIPA) buffer, and the protein concentration was determined using the BCA method. Following normalization and electrophoretic separation, proteins were transferred onto polyvinylidene fluoride (PVDF) membranes. The membranes were blocked with 5% skim milk for 1 h at room temperature and then incubated overnight at 4 °C with primary antibodies against TSG101 (NB200-112, Novus Biologicals, USA) and CD63 (NBP2-32830, Novus Biologicals, USA). Subsequently, the membranes were washed with Tris-buffered saline with 0.1% Tween 20 (TBST) and incubated with corresponding horseradish peroxidase (HRP)-labeled secondary antibodies antibody (31430, Invitrogen, USA) for 2 h at room temperature. After further washing with TBST, the signals were detected and visualized using an enhanced chemiluminescence (ECL) solution and ChemiDoc TM imager (FluorChemE, Germany). Image analysis was performed using ImageJ software (Ver. 1.53, National Institutes of Health USA, http://imagej.nih.gov/ij).

### Enzyme-linked Immunosorbent Assay (ELISA)

To quantify the level of sFlt-1 and PlGF in urine samples, ELISA was performed using Human sFLT-1 / sVEGFR1 ELISA Kit (Arigo biolaboratories, ARG81424) and Human PlGF ELISA kit (abcam, ab260056). Urinary exosomes, which were separated using BEST, were added to each well of a microplate following the manufacturer’s instructions. The absorbance at 450 nm was measured using the Synergy HTX multi-mode reader (BioTek instruments). The concentration of the target protein was determined by the standard curve (Fig. 4 B and C).

### Statistical analysis

Statistical analysis was conducted using Microsoft Excel. Descriptive statistics, including means and standard errors (S.E.), were calculated for the data. The independent two-tailed t-test with a significance level of α = 0.05 was performed to compare the variables between two groups: healthy controls (*n* = 3) and patients with preeclampsia (*n* = 3). Additionally, a paired t-test was performed to compare the variables within each group. By employing both independent and paired t-tests, differences between groups and within groups were comprehensively evaluated, ensuring a thorough analysis of the data.

## Supplementary Information

Supplementary Material 1

## Data Availability

All data are available in the main text or the supporting information.
