# Supplementary material for: Understanding the role of soluble proteins and exosomes in non-invasive urine-based diagnosis of preeclampsia
Source: Sci Rep. 2024 Oct 15;14:24117. doi: 10.1038/s41598-024-75080-2 (PMC11482518; doi:10.1038/s41598-024-75080-2)
Supplement: Supplementary file 1 — Supplementary Material 1 [file 41598_2024_75080_MOESM1_ESM.pdf]

Supplementary information

Understanding the Role of Soluble Proteins and Exosomes in Non-Invasive Urine-Based Diagnosis of Preeclampsia

Taewoon Kim[1†], Harshitha Kallubhavi Choodinatha[2, 3, 4†], Kwang Sik Kim[1], Kyusoon Shin[1], Hyeon Ji Kim[2, 3, 4], Jee Yoon Park[2, 3, 4*], Jong Wook Hong[1, 5, 6*], and Luke P. Lee[7-11*]

[1]Department of Bionanotechnology, Graduate School, Hanyang University, Seoul 04763, Korea.

[2]Department of Obstetrics and Gynecology, Seoul National University College of Medicine.

[3]Seoul National University, Seoul, Korea.

[4]Department of Obstetrics and Gynecology, Seoul National University College of Medicine, Seoul National University Bundang Hospital, Seongnam, Korea.

[5]Department of Medical and Digital Engineering, Graduate School, Hanyang University, Seoul 04763, Korea.

[6]Department of Bionanoengineering, Hanyang University, Gyeonggi-do 15588, Korea.

[7]Harvard Medical School, Harvard University; Department of Medicine, Brigham and Women's Hospital, Boston, Massachusetts, USA.

[8]Department of Bioengineering, University of California at Berkeley, Berkeley, CA, USA.

[9]Department of Electrical Engineering and Computer Science, University of California at Berkeley, Berkeley, CA, USA.

[10]Department of Biophysics, Institute of Quantum Biophysics, Sungkyunkwan University, Suwon, Korea.

[11]Department of Chemistry & Nanoscience, Ewha Womans University, Seoul, Korea.

[†]Taewoon Kim and Harshitha Kallubhavi Choodinatha contributed equally to this study as co-first authors.
*Jee Yoon Park, Jong Wook Hong, and Luke P. Lee contributed equally to this study as co-corresponding authors.

*Corresponding authors: jyparkmd08@snu.ac.kr (J.Y. Park); jwh@hanyang.ac.kr (J.W. Hong); lplee@bwh.harvard.edu (L. P. Lee)

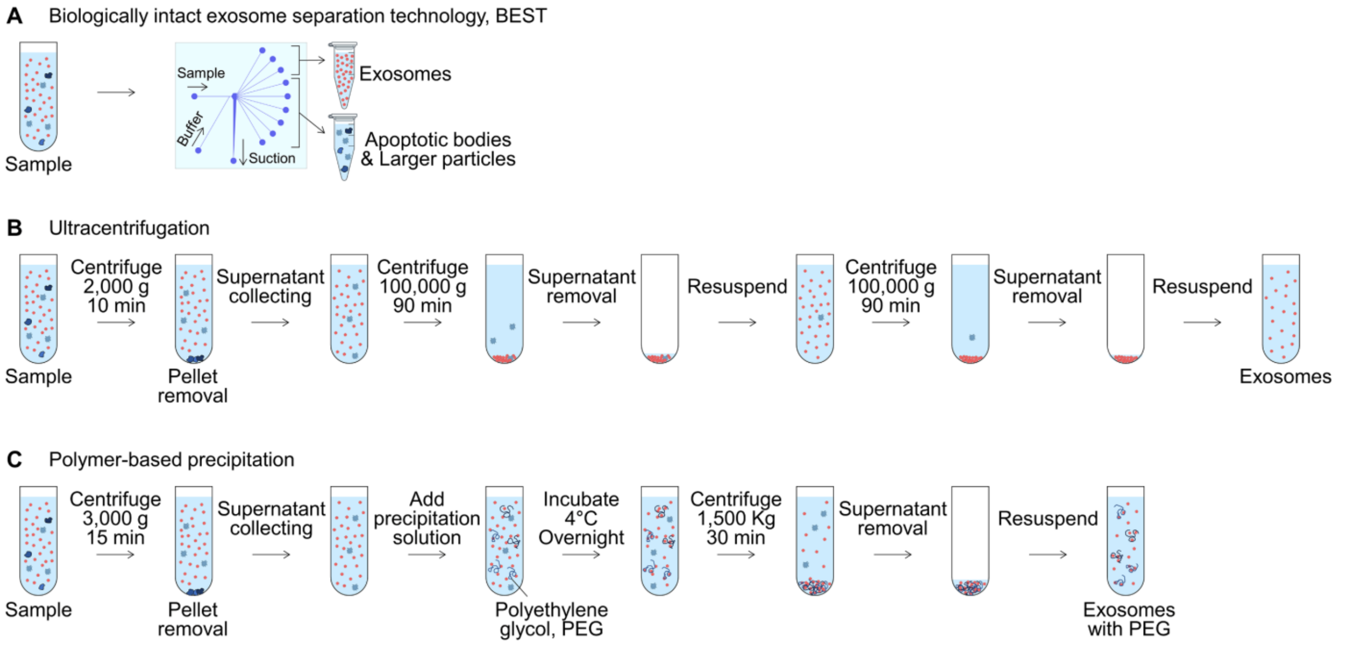

Supplementary Fig. 1 Operation steps of different separation techniques of exosomes. (A) Biologically intact exosome separation technology (BEST) separates particles by amplifying the difference in their center of gravity through geometric structure and manipulation of flow rates. (B) Ultracentrifugation separates exosomes using powerful centrifugal forces generated at high rotational speeds. (C) Precipitation involves aggregating exosomes by creating a network with polyethylene glycols.

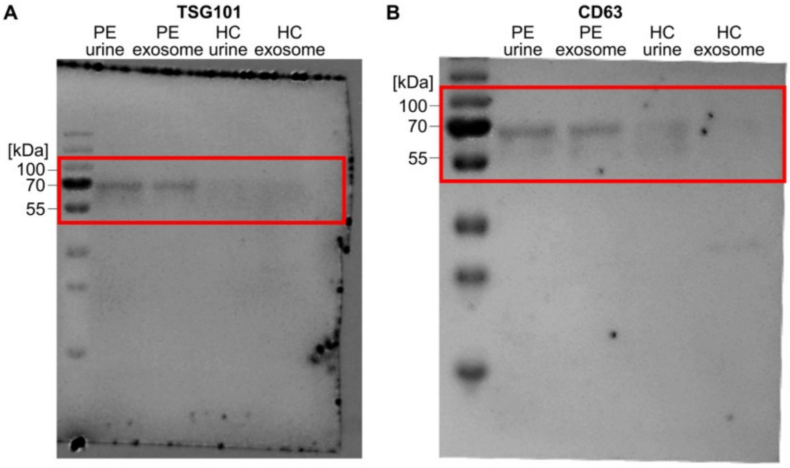

Supplementary Fig. 2 The uncropped images of the western blot for exosomal markers. (A) Expression of TSG101 in urine and urinary exosomes from PE and HC. (B) Expression of CD63 in urine and urinary exosomes from HC and PE. All cropped images are shown in Fig. 2. PE, patients with preeclampsia; HC, healthy controls.
